# Hysteroscopic management of retained products of conception: A single center observational study

**Published:** 2020-01-24

**Authors:** L Alonso Pacheco, D Timmons, M Saad Naguib, J Carugno

**Affiliations:** Gynecology Endoscopy Unit. Centro Gutenberg. Malaga, Spain;; Department of Obstetrics, Gynecology and Reproductive Sciences. University of Miami. Miller School of Medicine. Miami FL. USA

**Keywords:** Hysteroscopy, Retained Products of Conception, Gutenberg Classification, Doppler Ultrasound

## Abstract

**Background:**

Retained products of conception (RPOC) are defined as the presence of tissue inside the uterine cavity after delivery or termination of a pregnancy. Operative hysteroscopy is associated with increased surgical success and decreased postoperative formation of intrauterine adhesions. The aim of this study is to report our experience in hysteroscopic management of RPOC.

**Methods:**

A retrospective chart review identified patients who underwent hysteroscopic removal of retained products of conception at a single center (n=45). Basic demographic data, surgical findings and applied technique were reviewed. Chi Square and independent samples t-tests were performed when appropriate. A significance level of p<0.05 was accounted.

**Results:**

Of all cases included, 64% were the result of a spontaneous or elective abortion and 47% were from patients who had failed previous treatment. Previous medical or surgical treatment was observed in 37.9% of patients labeled as type 0-1 versus 62.5% of type 2-3 (p=0.1138). The timing between the end of the preceding pregnancy and hysteroscopic removal was in average 2.62 months in type 0-1 compared to 1.7 months in type 2-3 (p=0.1068). All patients who were classified as type 2-3 required the use of monopolar energy during the surgery, compared to zero patients who were classified as type 0-1 (p < 0.0001).

**Conclusion:**

Operative hysteroscopy remains a safe and highly effective option for the management of RPOC and should be the preferred method compared to traditional dilatation and suction curettage.

## Introduction

Retained products of conception (RPOC) are defined as the presence of any tissue (placental or fetal) that remains inside the uterine cavity after delivery or termination of a pregnancy (Hoveyda and MacKenzie, 2001). RPOC complicates 1% of all pregnancies and can be seen following medical or surgical termination, spontaneous abortion, vaginal or cesarean delivery (Westendorp et al., 1998, van den Bosch et al., 2008). Patients with RPOC most commonly presenting vaginal bleeding and RPOC are considered one of the most frequent causes of post-partum hemorrhage (PPH) (Sellmyer et al., 2013). Patients can also present with amenorrhea, pelvic pain, fever or abnormal vaginal discharge (Hakim-Elahi et al., 1990). Most patients with RPOC present with vaginal bleeding within days to weeks following pregnancy resolution, however, RPOC have been found to persist in patients for multiple years (Swan and Woodruff, 1969; Dyer and Bradburn, 1971).

**Figure 1:**
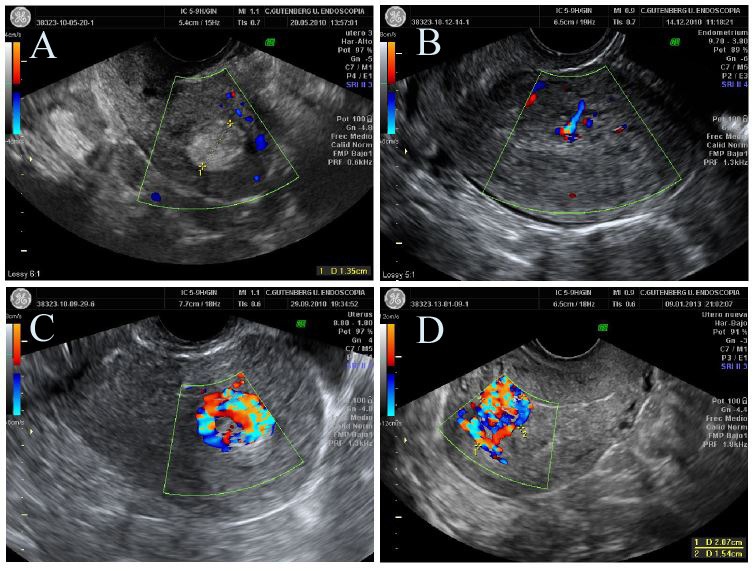
Ultrasonographic patterns of RPOC. Gutenberg Classification. A- Type 0: hyperechogenic avascular mass. B-Type 1: Different echoes with minimal or no vascularization. C- Type 2: Highly vascularized mass confined to the cavity. D- Type 3: Highly vascularized mass with highly vascularized endometrium.

The diagnosis of RPOC can be challenging. It relays on the patient’s clinical presentation, laboratory results and most importantly, ultrasound findings. Ultrasound is the imaging modality of choice when RPOC are suspected. The presence of an endometrial mass is the most sensitive finding for RPOC diagnosis, the absence of a mass essentially excluding RPOC from a differential diagnosis (Durfee et al., 2005). Kamaya et al. (2009) evaluated ultrasound images of patients with suspected RPOC and categorized them into one of four types based on Doppler imaging features (Figure 1).

**Figure 2:**
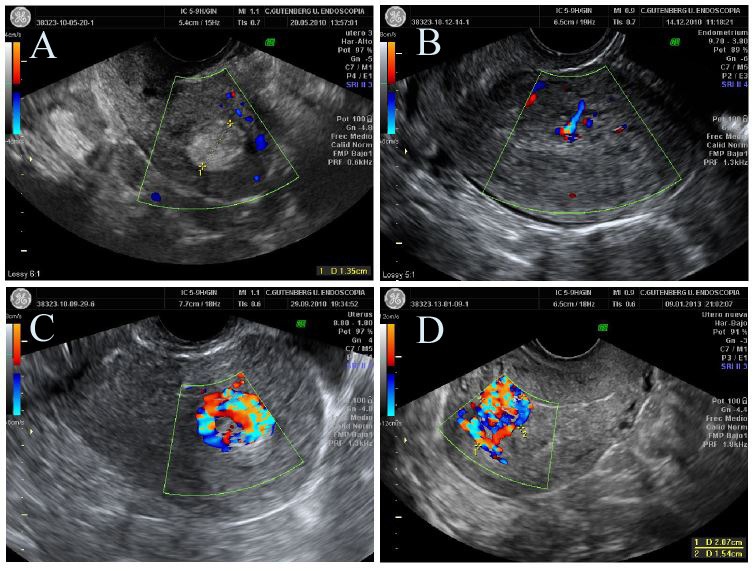
Hysteroscopic patterns of RPOC. Gutenberg classification. A- Type 0: white mass in with no clear structures. B- Type 1: well-defined avascular chorionic villi. C- Type 2: Well Vascularized chorionic villi. D- Fig 4: Aneurism over myometrium in the implantation area.

This was the first attempt to classify RPOC based on Doppler vascularity. The four types ranged from Type 0 (avascular) to type 3 (marked vascularity). This Doppler characterization was then adapted to create the Gutenberg Classification of RPOC which incorporated, both, vascularity and echogenicity of ultrasound findings (Table I) (Tinelli and Haimovich, 2017) (Figure 2).

**Table I:** — Gutenberg Classification: Ultrasonographic patterns of RPOC (Tinelli and Haimovich, 2017).

Type 0: Hyperechogenic avascular mass
Type 1: Different echoes with minimal or no vascularity
Type 2: Highly vascularized mass confined to the cavity
Type 3: Highly vascularized mass with highly vascularized myometrium

RPOC can be treated either medically or surgically. The choice of treatment is made upon a patient’s clinical presentation and the physician’s preference. Typically, surgical management involves dilation and suction curettage (D&C) which has associated risks of uterine perforation, infection, and development of adhesions with subsequent infertility (Ikhena, et al., 2016). While a D&C can be performed under ultrasound guidance, no direct intrauterine visualization of the cavity is possible.

The procedure is considered ‘blind’ and is associated with an up to 30% chance of developing intrauterine adhesions (IUA) (Yu et al., 2008). According to Yu et al. (2008) the most important step to prevent IUA and the possible development of Asherman’s Syndrome is to avoid post-partum or post-abortion curettage. When possible, operative hysteroscopy should be considered for surgical management as it has been shown to be both effective at diagnosing and treating RPOC (Tchabo, 1984; Goldenberg et al., 1997).

Operative hysteroscopy has many advantages compared to traditional D&C and has continued to grow in popularity for management of RPOC. The use of operative hysteroscopy allows for direct visualization of the uterine cavity enabling the surgeon to remove RPOC in a precise and targeted fashion, and it allows for visual confirmation of complete removal of RPOC (Smorgick et al., 2017). Compared to traditional D&C, operative hysteroscopy was found to be significantly superior in achieving complete uterine evacuation with persistence of RPOC seen in only 1.4% of cases treated hysteroscopically compared to 28.8% of cases treated with traditional blind suction D&C. It also showed that the use of operative hysteroscopy is associated with a significantly less likelihood of developing IUA (12.8% vs. 29.6%, p <0.001) (Hooker et al., 2016).

The aim of this study is to report our experience in hysteroscopic management of RPOC adopting the Gutenberg Classification for RPOC. The ultrasound/hysteroscopy imaging correlation of RPOC will be provided.

## Materials and methods

### Patients

Institutional Review Board approval was obtained. A retrospective chart review was performed at a single center in a large urban European hospital (Gutenberg Center, Malaga, Spain). A search was performed to identify all patients who underwent hysteroscopic removal of RPOC from November 1 st 2008 to December 31 st 2017. The procedure was performed by a single surgeon on a total of 45 patients. All medical records were reviewed. Collected data included basic demographics, obstetric history, preceding pregnancy outcome, previous treatments, and time between initial management and surgical management (Table II). Preoperative ultrasound images were reviewed using the Gutenberg Classification for RPOC. Hence, patients were placed into one of the two groups based on ultrasound findings: Type 0 and 1 (limited to no vascularization group) were combined and compared against type 2 and 3 (moderate to severe vascularization group) (Table III). All operative reports were evaluated to identify the cases that required the use of monopolar electrocautery to provide hemostasis.

**Table II:** — Demographic data (n = 45).

Age, years (mean, SD, range)		35.9 ± 4.5 (26-45)
Obstetric History		
	Previous NSVD	15 (33.3%)
	Previous C/S or Myomectomy	16 (35.6%)
	Previous Spontaneous or Elective Abortion	29 (64%)
Pregnancy Preceding RPOCs		
	Abortion	29 (64.4%)
	Vaginal	6 (13.3%)
	Cesarean	5 (11.1%)
	Unknown	5 (11.1%)
Time between delivery/abortion and surgery, months (mean, SD, range)		2.3 ± 1.8 (1-10)
Previous Treatment		
	None	24 (53.3%)
	D&C	11 (24.4%)
	Medical	10 (22.2%)

SD = Standard Deviation

**Table III:** — Frequency of RPOC based on Gutenberg Classification (n = 45).

Type 0	6 (13.3%)
Type 1	23 (51.1%)
Type 2	11 (24.4%)
Type 3	5 (11.1%)

### Operative Technique

**Figure 3:**
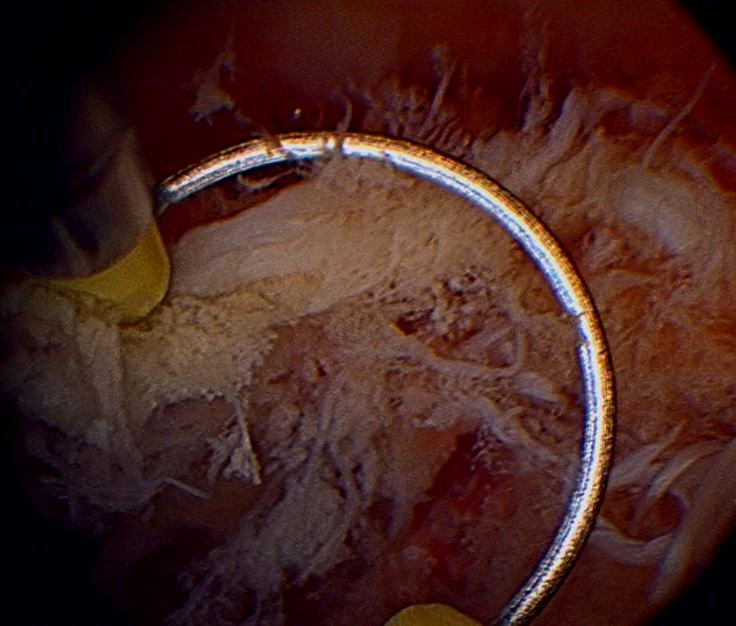
Detailed view of the loop used as curette.

Operative Hysteroscopy with removal of RPOC was performed in the operating room under general anesthesia in all cases. A 27 Fr. resectoscope (Karl Storz, Tüttlingen, Germany) with a cutting loop and glycine 1.5% as a distension medium was used. In all cases, cervical preparation was achieved with oral Misoprostol (400 mcg) the night before the procedure. When needed, additional cervical dilation was achieved using Hegar dilators of up to 10 mm. After introducing the hysteroscope, under direct visualization, the RPOC and their location within the endometrial cavity was documented. The loop of the resectoscope was then used as a curette (cold) to extract the remains in fragments without using electrocautery (Figure 3). An attempt was made to avoid electrocautery in all cases, however, in situations when difficulty was encountered completing total evacuation due to firm adherence of RPOC to the uterine wall with associated excessive bleeding, monopolar electrosurgery was used to provide hemostasis to allow for complete evacuation of the uterine cavity. After the procedure, a follow up visit was performed for confirmation of the symptoms with a clinical resolution at 6 weeks. Also, a pelvic ultrasound was performed within the first week after the first menstrual cycle following the evacuation, thus confirming the success of the intervention.

### Statistics

All statistical analyses were performed using SPSS for Microsoft Windows (SPSS Statistical Software V.22.0, IBM, Armonk, NY). Chi Square and independent t-tests samples were performed with a significance level of P<0.05.

## Results

The average age was 35.9 +/- 4.5 years old. The preceding pregnancy resulting in RPOC was most commonly an abortion (spontaneous or elective) comprising 64.4% of all cases (n=29). RPOC were less commonly seen following vaginal delivery (13.3%, n=6) and cesarean deliveries (11.1%, n=5). In five reviewed charts (11.1%) the type of preceding pregnancy was lacking. With these details the resolution type could be listed.

On average, the procedure was performed 2.2 +/- 1.8 months after completion of the preceding pregnancy. Twenty-four (53.3%) patients did not previously receive any treatment, eleven (24.4%) previously had blind suction D&C, and ten (22.2%) had received medical treatment (methergine or misoprostol). No patient previously underwent hysteroscopic resection.

Further evaluation of the groups created upon Gutenberg Classification showed no significant demographic differences. Also, previous treatments, timing between preceding pregnancy and operative data were compared (Table IV). From these comparisons, 37.9% of patients labeled as type 0-1 had previous medical or surgical treatment compared to 62.5% of type 2-3 (p=0.1138). The timing between the end of the preceding pregnancy and hysteroscopic removal was 2.62 months in type 0 - 1 compared to 1.7 months in type 2-3 (p=0.1068). Interestingly, all patients who were classified as type 2 - 3 required the use of monopolar energy during surgery, compared to zero patients who were classified as type 0 - 1 (p<0.0001).

**Table IV:** — Differences based on Gutenberg Classification.

	Type 0 or 1 (n = 29)	Type 2 or 3 (n = 16)	p
Age (yrs), mean ± SD	36.7 ± 4.3	34.6 ± 4.5	0.13
Use of monopolar energy	0% (0/29)	100% (16/16)	<0.0001
Previous treatment (medical or surgical)	37.9% (11/29)	62.5% (10/16)	0.1138
Time between delivery/abortion and surgery (months), mean ± SD	2.62 ± 2.1	1.70 ± 1.2	0.1068

SD = Standard Deviation

No intraoperative complications were noted. All patients had a followed-up visit 6 weeks after the procedure in which confirmation of the clinical resolution of symptoms was obtained.

Finally, transvaginal ultrasound was performed to confirm the absence of RPOC in the uterine cavity in all patients.

## Discussion

**Figure 4:**
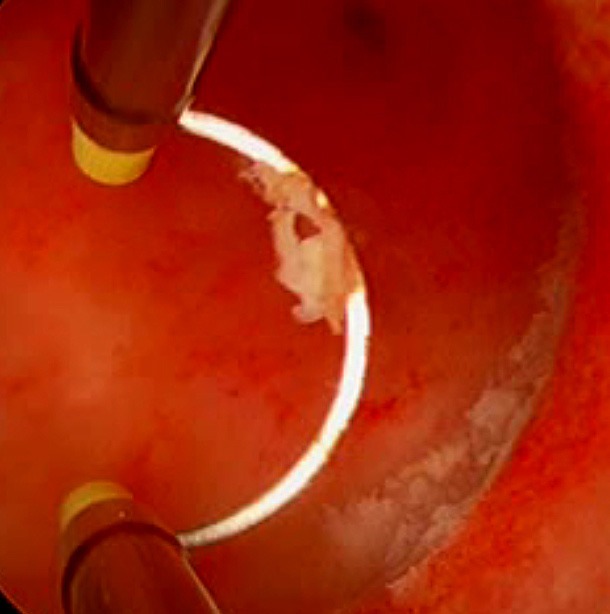
Implantation area after resection.

Our study appears to be the first to investigate the use of hysteroscopic management of RPOC incorporating the Gutenberg Classification for a preoperative classification with the aim to predict the risk of intraoperative bleeding during hysteroscopic removal of RPOC. During our study, we found that hysteroscopic removal of RPOCs was a safe and highly successful procedure, with no surgical complications encountered. Plus, all patients were reported to have complete removal of RPOC postoperatively (Figure 4).

This study further contributes to the existing literature on the efficacy of operative hysteroscopy for surgical management of RPOC. Operative hysteroscopy remains the gold standard for diagnosis and management of intrauterine pathology by consistently showing to be the best surgical procedure when encountering RPOC.

Also, this study demonstrates the effort to find an efficient preoperative classification that will allow the surgeon to predict the risk of bleeding during hysteroscopic removal of RPOC. This could be further used to assist the surgeon on the decision of the location of the procedure, thus, allowing the surgeon to determine the safest environment in which to safely perform the procedure (in office vs operating room setting).

An important finding from this study is that all patients classified as Gutenberg type 2 or 3 RPOC required the use of monopolar energy during the procedure, compared to none of the patients classified as type 0 - 1. To an extent, this seems logical as type 2 and 3 RPOC are classified in such characteristically setting of increased vascularity and echogenicity on ultrasound. In cases of minimal to no vascularity on ultrasound (Type 0 - 1), the use of the resectoscope as a ‘cold’ curette without energy, was associated with complete evacuation of the cavity with no reported complications. Nevertheless, minimal bleeding is typically encountered, and the retained tissue typically detaches easily from the uterine wall. This contrasts with type 2 - 3, where an attempt was made to remove RPOC without energy, however, firm adherence of RPOC or active bleeding required the use of monopolar energy. Despite the use of energy, no surgical complications were noted, and complete resection of the RPOC was demonstrated on post-operative ultrasound.

While not statistically significant in our small sample size, patients classified as type 2-3 had a shorter time between the end of their pregnancy and need for hysteroscopic intervention (1.7 months compared to 2.6 months). Type 2 - 3 are associated with more vascularity, and these patients are likely to reach their physicians earlier in the setting of earlier onset or heavier vaginal bleeding compared to patients with type 0-1. Also, patients with type 2-3 frequently had and failed some form of previous treatment compared to type 0 - 1 (62% vs 37.9%). As our study demonstrated, in many cases, monopolar energy was needed to treat type 2 - 3 RPOC because of firm adherence. It is likely that this increased adherence to the uterine wall resulted in more patients with type 2 – 3, failing traditional treatment.

As stated above, ultrasound remains the gold standard for imaging when RPOC are suspected. The use of the Gutenberg Classification for RPOC is not only crucial for surgical planning but is also important for patient counseling. When the diagnosis of RPOC is suspected, if properly counseled, expectant management is an acceptable option and the patient has minimal to mild symptoms. However, if ultrasound imaging is consistent with Gutenberg Type 2 or 3 RPOC, the patient should be educated on the presence of the increased vascularity and that earlier and heavier vaginal bleeding has been seen in patients with this finding. Similarly, patients classified as Gutenberg Type 2 or 3 should be counseled that while traditional treatments are acceptable, a higher percentage of patients fail these traditional treatments and ultimately require hysteroscopic resection. Providing patients with appropriate counseling allows for shared decision making to occur and hopefully avoids unnecessary or harmful interventions.

Although operative hysteroscopy for management of RPOC has clearly been demonstrated to be the superior method of surgical intervention, traditional D&C remains the most common form of treatment for RPOC. Disadvantages of performing a blind curettage to evacuate RPOC include increased risk of uterine perforation, development of intrauterine adhesions, pelvic infection and incomplete evacuation with persistence of RPOC, thus, with the need for repeat treatment. Hysteroscopic management of RPOC reduces all risks associated with traditional D&C and is more effective at achieving complete evacuation on the first attempt. The use of hysteroscopy not only significantly reduces the risk of uterine perforation and formation of IUAs, but it also significantly reduces the risk of needing a repeat procedure in the setting of persistent RPOC.

The strength of this study is that it is the first to compare the use of hysteroscopic resection of RPOC in Gutenberg type 0 - 1 vs type 2 - 3 RPOC.

The use of both Doppler vascularity and echogenicity of ultrasound appearance is a valuable method to anticipate the need for electrocautery to coagulate focal bleeding or assist with adherent RPOC. Using this preoperative categorization will assist surgeons to anticipate findings alike and hence to be prepared with the adequate equipment, necessary to successfully and safely complete the case.

Importantly, we acknowledge limitations such as a small sample size and retrospective nature of the study which limits the quality of data. In addition, the use of monopolar energy and glycine as distention media are worth mentioning. Indeed, although in our study we reported no complications due to the use of monopolar energy, neither fluid overload nor any complications related to use of Glycine as distention media, starting from January 2018, our hospital transitioned to hysteroscopic instruments with bipolar energy, considered safer than monopolar energy, thus allowing the use of normal saline as distention media with lower risk of distention media related complications. We strongly recommend the use of bipolar instruments and normal saline as distention media when performing hysteroscopic removal of retained products of conception. Finally, we used orally dispensed Misoprostol (400mcg) the night before for cervical ripening. Misoprostol has been demonstrated to be an effective means of cervical ripening prior to hysteroscopy, and while we chose an oral route, both, buccal and vaginal routes of administration have been shown to provide adequate cervical ripening (Hua et al., 2016).

## Conclusions

Operative hysteroscopy remains a safe and highly effective option for the management of RPOC and should be used instead of traditional D&C. Although, this series was conducted using monopolar energy hysteroscopic resectoscope and Glycine 1.5% as distention media, the use of bipolar instruments or hysteroscopic morcellators is currently recommended as safer alternatives. In patients with type 2 - 3 RPOC, as classified by the Gutenberg Classification, hysteroscopic management is an effective and safe treatment modality. Preoperative ultrasound findings and the use of the Gutenberg Classification will help physicians counsel patients more effectively and help surgeons to be more prepared with the necessary equipment available in the operating room. Although more data and larger studies are needed, we believe this study can provide surgeons sufficient reasons for avoiding traditional D&C and choose hysteroscopic resection with or without the use of electrocautery when encountered with RPOC.
